# Case report: Lympho-histiocytic meningoencephalitis with central nervous system vasculitis of unknown origin in three dogs

**DOI:** 10.3389/fvets.2022.944867

**Published:** 2022-08-24

**Authors:** Isabel Zdora, Jonathan Raue, Franz Söbbeler, Andrea Tipold, Wolfgang Baumgärtner, Jasmin Nicole Nessler

**Affiliations:** ^1^Department of Pathology, University of Veterinary Medicine Hannover, Hannover, Germany; ^2^Center of Systems Neuroscience, Hannover, Germany; ^3^Department for Small Animal Medicine and Surgery, University of Veterinary Medicine Hannover, Hannover, Germany

**Keywords:** meningoencephalitis of unknown origin (MUO), central nervous system (CNS), sterile, canine (dog), inflammation, brain

## Abstract

Meningoencephalitis of unknown origin (MUO) is an umbrella term for a variety of subtypes of meningoencephalitis of dogs and cats with no identifiable infectious agent. In dogs, granulomatous meningoencephalitis (GME), necrotizing meningoencephalitis (NME), and necrotizing leukoencephalitis (NLE) are the most commonly reported subtypes. However, sporadically there are reports about other subtypes such as greyhound encephalitis or eosinophilic meningoencephalitis. The following case series presents three dogs with peracute to acute progressive signs of encephalopathy. The magnetic resonance imaging (MRI) of two dogs (*post mortem n* = 1/2) showed severe, diffuse swelling of the cortical gray matter with increased signal intensity in T2weighted (w) and fluid-attenuated inversion recovery (FLAIR) and decreased signal intensity in T1w. Additionally, focal to multifocal areas with signal void in both dogs and caudal transforaminal herniation of the cerebellum in one dog was observed. *Post mortem* histopathological examination revealed lympho-histiocytic encephalitis and central nervous system (CNS) vasculitis in all dogs. No infectious agents were detectable by histopathology (hematoxylin and eosin stain), periodic acid-Schiff reaction (PAS), Ziehl-Neelsen stain and immunohistochemistry for Canine adenovirus-1, Parvovirus, *Listeria monocytogenes*, Parainfluenzavirus, *Toxoplasma gondii*, Herpes-suis virus, Pan-Morbillivirus, Tick born encephalitis virus, Severe acute respiratory syndrome coronavirus (SARS-CoV) 2. Furthermore, two dogs were tested negative for rabies virus. To the best of the authors' knowledge, this is the first report of a lympho-histiocytic encephalitis with CNS vasculitis with no identifiable infectious agent. It is suggested to consider this as an additional subtype of MUO with severe clinical signs.

## Introduction

Meningoencephalitis of unknown origin (MUO) is an umbrella term for a variety of subtypes of meningoencephalitis of dogs where no infectious agent can be identified (1–6). Granulomatous meningoencephalitis (GME), necrotizing meningoencephalitis (NME), and necrotizing leukoencephalitis (NLE) of dogs are the most commonly reported histopathological subtypes (3, 7–10). Other less commonly reported subtypes of MUO include eosinophilic meningoencephalitis, and greyhound encephalitis (11–13). The specific etiopathology of MUO is unknown so far, but a multifactorial pathology is suspected, involving an underlying - suspected mostly genetic - immunological defect (8, 14) and possible environmental triggers, for example an infectious or toxic agent (1–6). MUO is typically treated with anti-inflammatory or immunosuppressive drugs with varying prognosis depending on the subtype of MUO (4). A diagnosis is usually based on clinical signs, diagnostic imaging findings, cerebrospinal fluid examination, and exclusion of possible infectious agents. (1–4, 6, 8). However, a definitive diagnosis and especially the determination of the MUO subtype requires histopathologic examination (15). The known MUO subtypes present with distinct histopathological features. GME is characterized by an angiocentric, lymphocytic and granulomatous inflammation of the CNS (16). Here, inflammatory cell infiltrates are most often found within the white matter (17). In NME and NLE, lesions compromise CNS necrosis as well as lympho-histiocytic, often perivascularly located inflammation (17). In NME, the predilection site is the cerebral cortex, while in NLE, the white matter is primarily affected (17). Idiopathic eosinophilic meningoencephalitis shows necrosis and inflammatory infiltrates consisting of eosinophils and macrophages within the cerebral cortex (12). Greyhound encephalitis is considered a breed-associated MUO, which presents with non-suppurative inflammatory changes mainly found in the frontal lobe and olfactory bulb bilaterally (13). Furthermore, a large number of cases of MUO have not been specified as a distinct subtype (18, 19). None of the mentioned subtypes of MUO is typically accompanied by CNS vasculitis (12, 13, 16, 18, 19).

Vasculitis is defined as an inflammation of blood vessels with inflammatory cells infiltrating the damaged vascular wall as well as the perivascular space (20). Aside from primary vasculitis with no evident cause, secondary vasculitis due to different triggers represents the more common type reported (20, 21). Typical causes of secondary vasculitis include environmental noxae, reaction to different kinds of medication as well as hypersensitivity reactions (18). Vasculitis confined to the CNS is rarely reported (20, 22).

The present case series describes the macroscopic and histopathological findings of dogs that suffered from a so far undescribed meningoencephalitis with vasculitis restricted to the CNS. A causative infectious agent was not detectable.

## Materials and methods

All examinations were performed with written informed owner's consent according to ethical guidelines of the University of Veterinary Medicine Hannover, Germany, between 2017 and 2021.

Blood examinations were performed immediately after blood sampling and included blood cell count (ADVIA 120 Hematology System, Siemens Healthcare GmbH, Erlangen, Germany), biochemistry (cobas c 311 analyzer, Roche Deutschland Holding GmbH, Mannheim, Germany), and electrolytes (RAPIDLab 1260, Siemens Healthcare GmbH).

Radiography (Philips Bucky Diagnost, 2001, Hamburg, Germany, and AGFA CR85-X Digitalizer, 2007, Mortsel, Belgium) of the thorax in three planes was performed in case 1.

Magnetic resonance imaging (MRI; 3.0 T MRI scanner Achieva, Philips Medical Systems, Best, The Netherlands) of the brain was obtained under general anesthesia in one dog, and 20 min after euthanasia in another. MRI was not available in the third dog. After premedication with diazepam [0.5 mg/kg intravenously (i.v.)] and levomethadone with fenpipramide [0.2 mg/kg i.v. (L-Polamivet ^®^, MSD Tiergesundheit, Unterschleißheim, Germany)], anesthesia was inducted with propofol (dose to effect 1-3 mg/kg i.v.) followed by orotracheal intubation and connection to a semiclosed circle absorber system (Anesthesia ventilator, Cato^®^ Dräger, Germany). Anesthesia was maintained with isoflurane in an oxygen/air mixture (1:1, flow 50 ml/kg/min) in one dog (case 3). MRI was obtained in transversal, sagittal, and dorsal view in T2weighted (w) and T1w sequences pre and post contrast administration (gadoterate meglumine, 0.2mmol/kg i.v.). No contrast medium was applied in case 1. Fluid-attenuated inversion recovery (FLAIR) and gradient echo (GE) images were obtained in transversal plane. Cerebrospinal fluid (CSF) was sampled suboccipitally from the *cisterna magna post mortem* only in case 1 and was immediately examined for cell content *via* Fuchs-Rosenthal-chamber and microscopical cell differentiation. Case 1 and 3 were euthanized by i.v. administration of pentobarbital (100–357 mg/kg, Euthadorm^®^ CP-Pharma Handelsgesellschaft mbH, Burgdorf, Germany). A *post mortem* examination of all dogs was performed at the Department of Pathology, University of Veterinary Medicine Hannover, Germany. Following necropsy, specimens of various organs including brain, spinal cord, peripheral nerves, tonsil, lung, spleen, liver, heart, eye, thyroid gland, diaphragm, skeletal musculature, pituitary gland, adrenal glands, kidneys, urinary bladder, tongue, trachea, stomach, and small and large intestine were collected for further histological, histochemical, and immunohistochemical investigation. Samples were fixed in 10% neutrally buffered formalin for at least 24 h, paraffin wax-embedded, and cut into approximately 2 μm thick sections. Sections were stained with hematoxylin and eosin (HE) and central nervous system (CNS) regions with lesions were evaluated semi-quantitatively by using mild (single inflammatory cells in the perivascular space), moderate (1–3 layers of perivascular inflammatory cells), and severe (>3 layers of inflammatory cells in the perivascular space). Selected sections of CNS with inflammatory changes were further assessed by immunohistochemistry (IHC) and additional special stains including a periodic acid-Schiff reaction (PAS) and Ziehl-Neelsen stain. IHC for the detection of potential causative infectious agents was performed as described previously (18). This included using an anti-canine adenovirus-1 (DV4-1A; Custom Monoclonals International, Chris K. Grant, CA, USA, mouse monoclonal), anti-parvovirus (CPV1-2A1; Custom Monoclonals International, Chris K. Grant, CA, USA, mouse monoclonal), anti-listeria monocytogenes (DIFCO Laboratories, 2,469–563, rabbit polyclonal), anti-parainfluenzavirus (SV5-NP-C, Dr Randall, Department of Biochemistry and Microbiology, University of St Andrews, UK, mouse monoclonal), anti-toxoplasma gondii (Quartett, Cat. No. 201500102, rabbit polyclonal), anti-neospora caninum (Dr. Schares, Institute of Epidemiology, Friedrich-Loeffler-Institute, Federal Research Institute for Animal Health, Greifswald-Insel Riems, Germany, monoclonal mouse), anti-herpes-suis (Dr. Eskens, Veterinär-Untersuchungsamt Mittelhessen, Germany, polyclonal mouse), anti-pan-Morbillivirus (D110; kind gift from Prof. Dr. A. Zurbriggen, University of Bern, Switzerland, mouse monoclonal), anti-tick born encephalitis virus (K-D-3.BA; Prof. Holzmann, Department of Virology, University of Vienna, Austria, rabbit polyclonal) antibody. Additionally, IHC for the detection of severe acute respiratory syndrome coronavirus (SARS-CoV) 2 antigen using an anti-SARS CoV 2 nucleoprotein (NP) antibody (Sino Biological, 40143-MM05, mouse monoclonal) was performed on sections of brain and lung of all dogs using the Dako EnVision+ polymer system (Dako Agilent Pathology Solutions) and 3, 3'-Diaminobenzidine tetrahydrochloride (Sigma-Aldrich, St.Louis, MO, United States) as described previously (23, 24). Investigation of rabies-virus infection was performed in two dogs (case 2 and 3) at the Lower Saxony State Office for Consumer Protection and Food Safety. Furthermore, a set of immunological markers including anti-CD3 (Agilent Dako, Cat.No. A0452, rabbit polyclonal) for the detection of T-lymphocytes, anti-CD20 (Thermo Fisher Scientific, Cat. No. RB-9013-P, rabbit polyclonal) for the detection of B-lymphocytes as well as anti-CD204 (Abnova Corporation, Cat. No. MAB1710, mouse monoclonal) for the detection of macrophages was applied in IHC as described previously (25). In addition, Luxol Fast Blue (LFB) – Cresyl-Echt-Violet was performed to investigate myelin loss in all dogs.

## Cases

### Case 1

A 1.25 year old, male-neutered Chihuahua was presented with a four-day history of lethargy, decreased appetite, and tachypnea. He was regularly dewormed and vaccinated. In the general examination, the dog showed mild apathy and generalized high-frequency, low-amplitude tremor. Rectal body temperature was 38.7°C. Breathing pattern was normal, frequency was 32/min with physiological effort. Auscultation was inconclusive due to whole body tremor. Coughing and retching was provoked when palpating the larynx. On cardiac auscultation the heartbeat was regular with 120 beats per minute (bpm), no heart murmur was audible. Femoral pulses bilaterally were strong, regular, and synchronous with heart beat. Capillary refill time was under 2 s, the mucous membranes were pale-pink and moist. Peripheral lymph nodes were non-painful, soft and under 1cm in diameter on palpation. Abdominal palpation revealed no abnormal intraabdominal structures and no signs of pain. Mild serous ocular discharge in both medial canthus was noted without any other ocular abnormalities. Macroscopical evaluation of external ears, nose, and skin were unremarkable. Gait and posture were normal. At this timepoint, a neurological examination was not performed due to the lack of obvious involvement of the nervous system on general examination. Complete blood count, clinical chemistry, serum electrolytes, and abdominal ultrasonography were without clinically relevant abnormalities. The owner declined thoracic radiographs at this time point and decided for further outpatient therapy with non-steroidal anti-inflammatory and antibiotic medication. At home, 12 h after first presentation in the clinic, the dog developed generalized tonic-clonic seizure. The seizure had lasted for more than 2 h before the dog was presented to the emergency service again. The dog showed tonic-clonic movements, impaired consciousness, salivation, and a gurgling laryngeal stridor during in- and expiration. Body temperature was measured at 42°C, the heart rate was 162 bpm, no heart murmur was audible. Femoral pulses were strong bilaterally, regular, and synchronous with the heartbeat. Breathing frequency was 52/min. Capillary refill time was under 2 s, the mucous membranes were pale-pink and moist. The dog had brown-reddish diarrhea. Neurologic examination revealed generalized tonic-clonic seizure while the dog was in lateral recumbency. Menace response was absent in both eyes. The dog showed no facial paralysis and an increased tone of the masticatory muscles. Pupillary light reflex, gagging, strabismus, vestibulooccular reflexes, facial sensation, pain sensation and spinal reflexes were not evaluable due to ongoing seizure. The dog was stabilized with diazepam (4 × 2 mg/kg i.v., Diazepam Lipuro, B.Braun, Melsung, Germany) and phenobarbital (2 × 2 mg/kg i.v., Luminal, Desitin, Hamburg, Germany) and resuscitative fluid therapy (20 ml/kg/h for 3 × 20 min as bolus infusion, Sterofundin ISO Vetcare, B.Braun, Melsung, Germany) followed by continuous rate infusion (3 ml/kg/h, Sterofundin BG-5, B.Braun, Melsung, Germany). Thoracic radiographs were unremarkable. After cardiovascular stabilization and treatment of seizures leading to their interruption, neurological examination showed coma, bilateral non-responsive miotic pupils, and generalized absent cranial reflexes, while the breathing pattern remained normal, why a severe brainstem lesion with primary forebrain disease was suspected. Due to a grave prognosis, the owners elected for euthanasia.

**Figure 1:**
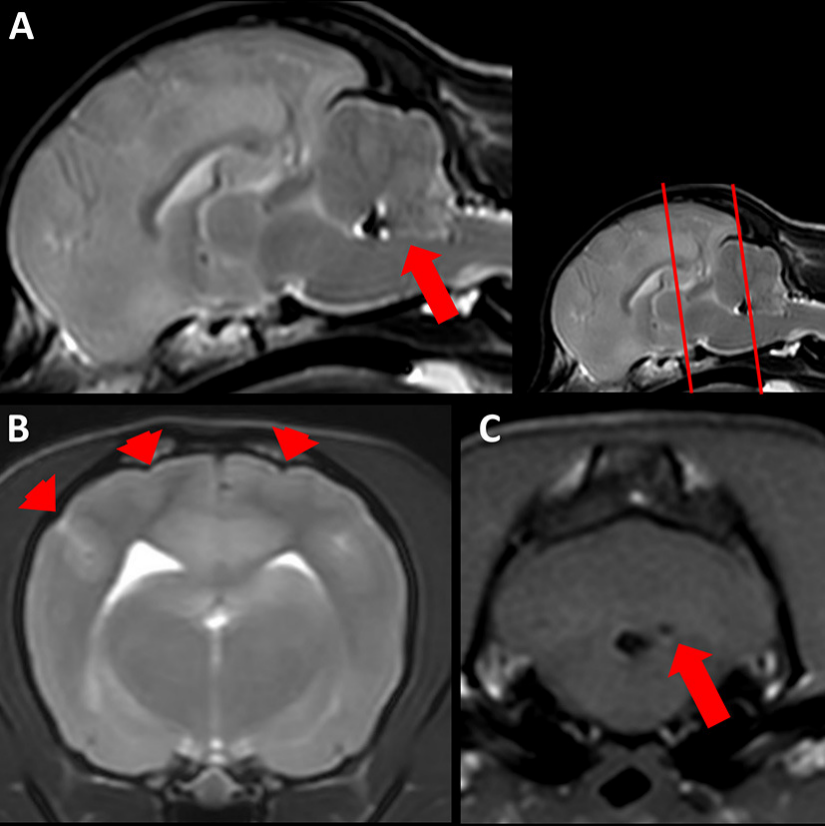
Magnetic resonance imaging (MRI) of a 1.25 year old Chihuahua (case 1). **(A)** Sagittal T2 weighted (w) *post mortem* MRI of the brain. Note the mild indentation of the rostral cerebellum due to increased volume of the cerebrum. Hypointense material fills the fourth ventricle (arrow), intraventricular hemorrhage is suspected. **(B)** Transversal T2w MRI of the cerebrum at the level of the caudal part of the hippocampus (level is indicated in the small inlay as the first red line). Note the generalized swelling of gray matter with flattened gyri and sulci (arrowheads). **(C)** Transversal T1w MRI of the cerebellum and brainstem (level is indicated in the small inlay as the second red line). Round, well demarcated intraaxial lesion with signal void (arrow), hemorrhage is suspected.

*Post mortem* MRI (Figure 1) showed generalized swelling of the gray matter in the cerebrum and cerebellum with secondary flattening of gyri and sulci. Cortical gray matter displayed increased signal in T2w and FLAIR and decreased signal in T1w. The boundary between subcortical white matter and cortical gray matter was mostly blurry in all sequences. Caudal brainstem and cerebellum showed multifocal, intraaxial, small, round lesions with signal void in T2w and T1w. The fourth ventricle and cisterna magna were filled with material causing signal void in T2w and T1w without significant mass effect.

Examination of CSF sampled *post mortem* atlanto-occipitally revealed severely elevated number of erythrocytes without signs of erythrophagocytosis. Leukocyte value was elevated, the exact number was not countable. Protein content measured 188 mg/dl (reference values <25 mg/dl). Cell differentiation showed 91% lymphocytes, 7% monocytes and 2% neutrophils.

**Figure 2:**
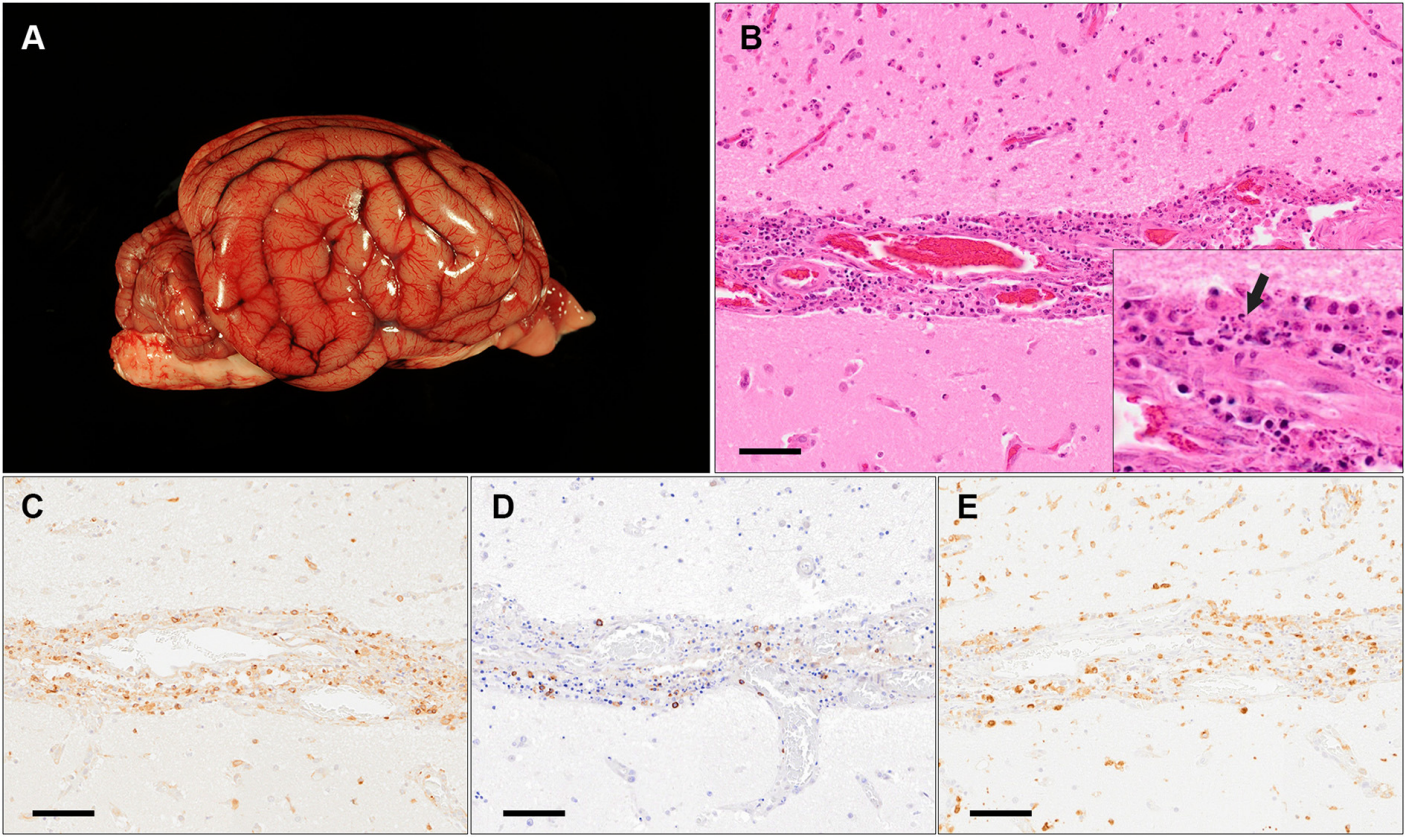
Macroscopic and histopathological findings of a 1.25 year old Chihuahua (case 1). **(A)** Macroscopic image of the brain with mild narrowing of sulci and flattening of gyri interpreted as edema. **(B)** Histopathology of the cerebral cortex at the level of the temporal lobe with moderate, leukocytoclastic vasculitis of the leptomeningeal blood vessels with fragments of degenerated inflammatory cells (arrow in insert) visible within the destructed vascular wall (HE stain; scale bar: 50μm). **(C–E)** Immunohistochemistry for CD3 **(C)**, CD20 **(D)** and CD204 **(E)** shows that the majority of infiltrating cells are comprised of T-lymphocytes [**(C)**; CD3-positive] and macrophages [**(E)**; CD204-positive] (Scale bars: 50 μm).

At necropsy, a moderate flattening of the gyri and narrowing of sulci of the brain (Figure 2A) was observed in conformity with the MRI results and interpreted as edema. In addition, mild cerebellar vermal herniation into the *foramen occipitale magnum* was visible. The meningeal vessels were moderately and diffusely congested. The dog had hemorrhagic intestinal content and a single nematode was found in the small intestine. Histopathologicallly, the gray matter of the cerebrum as well as the cerebellum showed moderate, multifocal, non-symmetrical, lympho-plasma-histiocytic, necrotizing inflammation. Inflammatory cell infiltrates were mainly found in the perivascular space. Furthermore, primarily small to medium sized blood vessels in the gray matter and especially prominent in the leptomeninx displayed loss of integrity of the vascular wall with moderate to severe infiltration with partially degenerated lymphocytes and macrophages resembling vasculitis of the leukocytoclastic type (Figure 2B). The spinal cord did not show any morphological changes.

IHC of selected sections of the CNS revealed infiltration of a moderate to high number of CD3-positive T-lymphocytes, low to moderate number of CD204-positive macrophages and few CD20-positive B-lymphocytes, respectively, in the partially destructed vascular wall and the perivascular space (Figures 2C–E). Using IHC and histochemistry, no infectious agent or signs of demyelination were detected.

In addition, mild, mucosal hemorrhages and few dilated crypts and mild flattening of villi were observed in the small intestine. Due to the macroscopic and histological lesion in the small intestine, parvovirus infection was ruled out by IHC. The spleen showed moderate lymphoid depletion. In the lung only moderate, diffuse, acute alveolar edema and hyperemia were found. These changes were interpreted as having developed during agony. No signs of parasites, e.g. *Angiostrongylus vasorum*, were found in the lungs, heart, gastrointestinal tract, or CNS via gross and microscopic examination. The remaining investigated tissues lacked significant microscopic lesions.

### Case 2

A 10 year old, male-neutered, medium sized, mixed breed dog was presented with a one-week history of progressive neurological signs. Initially, the dog was dull and showed a low head carriage. Clinical signs progressed to pacing in circles to the right, dysphoria, and suspected right sided visual deficits. There was no travel history. The dog was regularly vaccinated and dewormed.

The dog was presented in lateral recumbency. The dog was stuporous. Rectal body temperature was 38.2°C. Breathing pattern was normal, frequency was 34/min with physiological effort. Auscultation revealed mildly increased vesicular sounds, which were interpreted as still within physiological limits. On cardiac auscultation the heartbeat was regular with 90 bpm, no heart murmur was audible. Femoral pulses were strong, regular, and synchronous with heartbeat. Capillary refill time was under 2 s, the mucous membranes were pale-pink and mildly sticky on palpation. Macroscopical evaluation of eyes, external ears, nose, and skin were unremarkable.

The owner declined any further diagnostic attempt or therapy. After 3 h in the clinic, the dog suffered spontaneous cardio-respiratory arrest and died.

**Figure 3:**
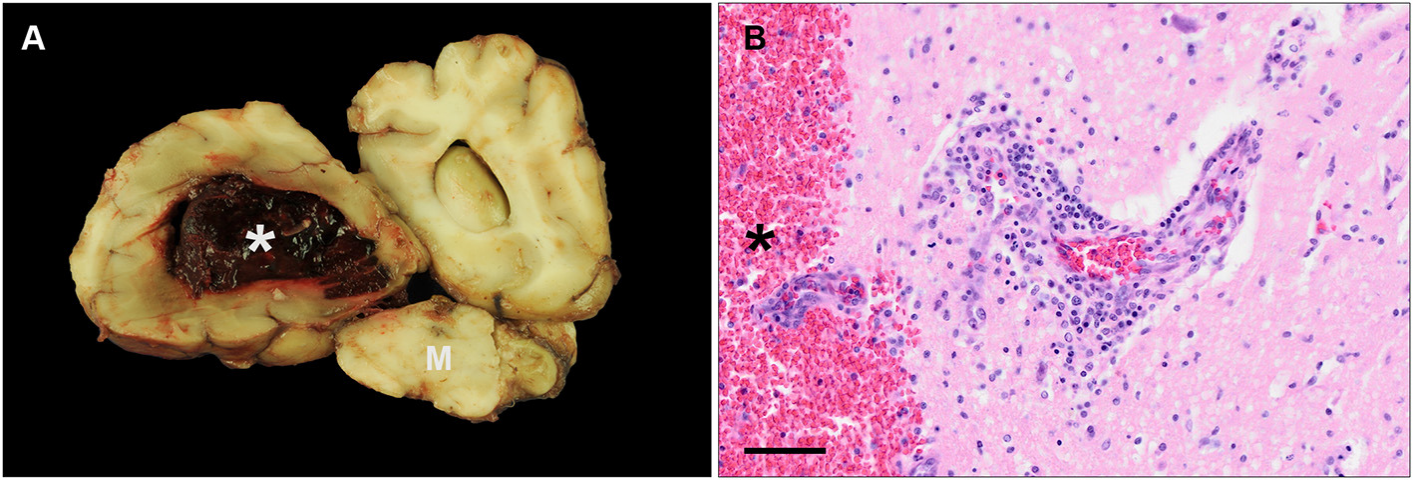
Macroscopic and histopathological findings of a 10 year old, mixed breed dog (case 2). **(A)** Macroscopic image of a transverse section of the cerebrum including the cerebral hemispheres and parts of the mesencephalon (M). The lateral ventricle of the left hemisphere displays severe, focal hemorrhage (asterisk). **(B)** Histopathology of the same area displayed in **(A)**. A blood vessel shows moderate vasculitis and perivascular infiltrates consisting of lymphocytes and macrophages. The lateral ventricle and adjacent parenchyma reveal hemorrhage (asterisk) (HE stain; scale bar: 50 μm).

Macroscopically, an approximately 4 cm in diameter sized accumulation of coagulated blood was found within the lateral ventricle of the left hemisphere of the brain (Figure 3A). The adjacent brain parenchyma showed multifocal hemorrhages. Other findings comprised mild endocardiosis of the atrioventricular valve as well as multiple nodular masses within the spleen. Histopathologically, focal, severe hemorrhage corresponding to the macroscopic finding was visible in the left lateral ventricle and the neighboring brain parenchyma. Furthermore, moderate to severe, lympho-histiocytic infiltrates were detected within the meninges and brain parenchyma, and associated with non-leukocytoclastic vasculitis, most frequently in the cerebral cortex, hippocampus, brain stem, and cerebellum (Figure 3B). Furthermore, similar, but mild inflammatory changes of leptomeningeal blood vessels were found in the cervical, thoracic, and lumbar spinal cord. Additionally, within the perineural tissue of the optic nerve of the left eye mild, lympho-histiocytic vasculitis was detected as well as a mild cataract. In between nerve fibers of the trigeminal nerve, moderate, focal, acute hemorrhage was found. Using IHC, perivascular infiltrates in the CNS comprised equal numbers of CD3- and CD20-positive lymphocytes and multifocal, irregular infiltration with few CD204-positive macrophages. Using IHC and histochemistry, no infectious agent or signs of demyelination were detected.

Further alterations of minor significance comprised mild, follicular hyperplasia in the tonsils and mild, multifocal anthracosis in the lung as well as mild, acute, diffuse, alveolar edema. The splenic nodules were diagnosed as nodular hyperplasia. No signs of parasites, e.g. *Angiostrongylus vasorum*, were found in the lungs, heart, gastrointestinal tract, or CNS via gross and macroscopic examination.

### Case 3

A 10.75 years old, male-neutered Australian Shepherd with a 2-day history of progressive gait abnormality and two self-limiting generalized tonic-clonic seizures was presented. Two months before presentation, the dog showed a left sided facial paralysis, which completely resolved after 15 days of prednisolone treatment by the primary veterinarian. The vaccination status was not reported.

At the time of presentation, the dog showed orofacial seizures, which developed into generalized tonic-clonic seizures and could be controlled with diazepam (2 mg/kg i.v.). Rectal body temperature was 39.4 °C. Heartbeat was 90 bpm without murmur on auscultation. Breathing pattern was normal, frequency was 40/min with physiological effort. Auscultation revealed mild vesicular lung sounds. Capillary refill time was under 2 s, the mucous membranes were pink and moist. Abdominal palpation was within normal limits. Macroscopical evaluation of eyes, external ears, nose, and skin were unremarkable. Blood examination revealed mildly elevated alanine aminotransferase (102 U/l; reference <50 U/l) and alkaline phosphatase (193 U/l; reference >150 U/l) activity. Clinical signs progressed within 24 hours, and the dog showed severe bradypnea and cyanosis. At neurological examination the dog was in lateral recumbency with generalized increased muscle tone and high-frequency, low-amplitude generalized tremor. He was comatose, showed bilateral absent palpebral reflexes and menace response and decreased pupillary light response on both eyes. Vestibuloocular and spinal reflexes were not tested. Consequently, the dog was intubated and mechanically ventilated.

**Figure 4:**
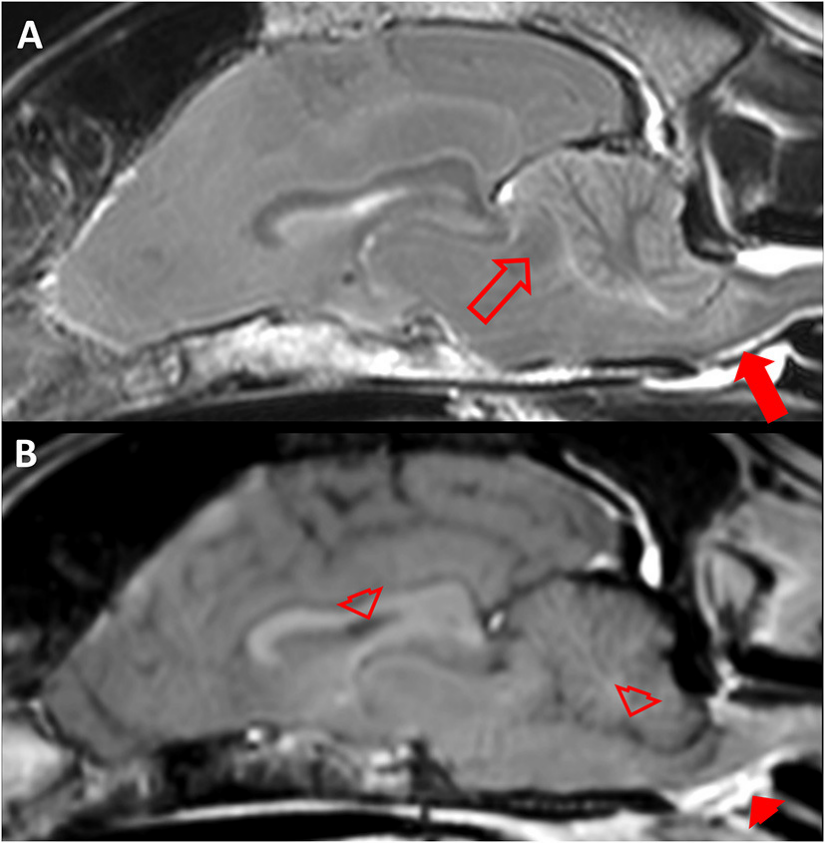
Magnetic resonance imaging (MRI) of a 10.75 year old Australian Shepherd (case 3). **(A)** Sagittal T2 weighted (w) MRI of the brain. Note the caudal cerebral herniation (empty arrow) which causes concave distortion of the rostral cerebellum and secondary transforaminal cerebellar herniation with compression of the brainstem and an intramedullary hyperintense lesion (filled arrow). **(B)** Sagittal T1w MRI approximately 3 min after intravenous contrast medium application. Note that there is physiological intravenous contrast enhancement extracranially (e.g., filled arrowhead) but no contrast medium is visible in structures which physiologically take up contrast medium (empty arrowheads).

Subsequent MRI (Figure 4) showed generalized flattened sulci and reduced volume of internal and external CSF space due to swelling of the CNS parenchyma. The FLAIR sequence revealed a subtle, diffuse, increased signal intensity of the internal capsule. There was transtentorial forebrain herniation as well as caudal foraminal herniation of the cerebellum with severe compression of the brainstem. At the site of compression, there was an intraaxial, T2w hyperintense lesion with multifocal signal void in the brainstem. There was no physiological contrast enhancement in the choroid plexus, which was suspected to be secondary to the compressed basilar artery preventing contrast agent to reach the CNS parenchyma.

Due to an infaust prognosis, no further examinations were performed and the dog was euthanized on owner's request.

**Figure 5:**
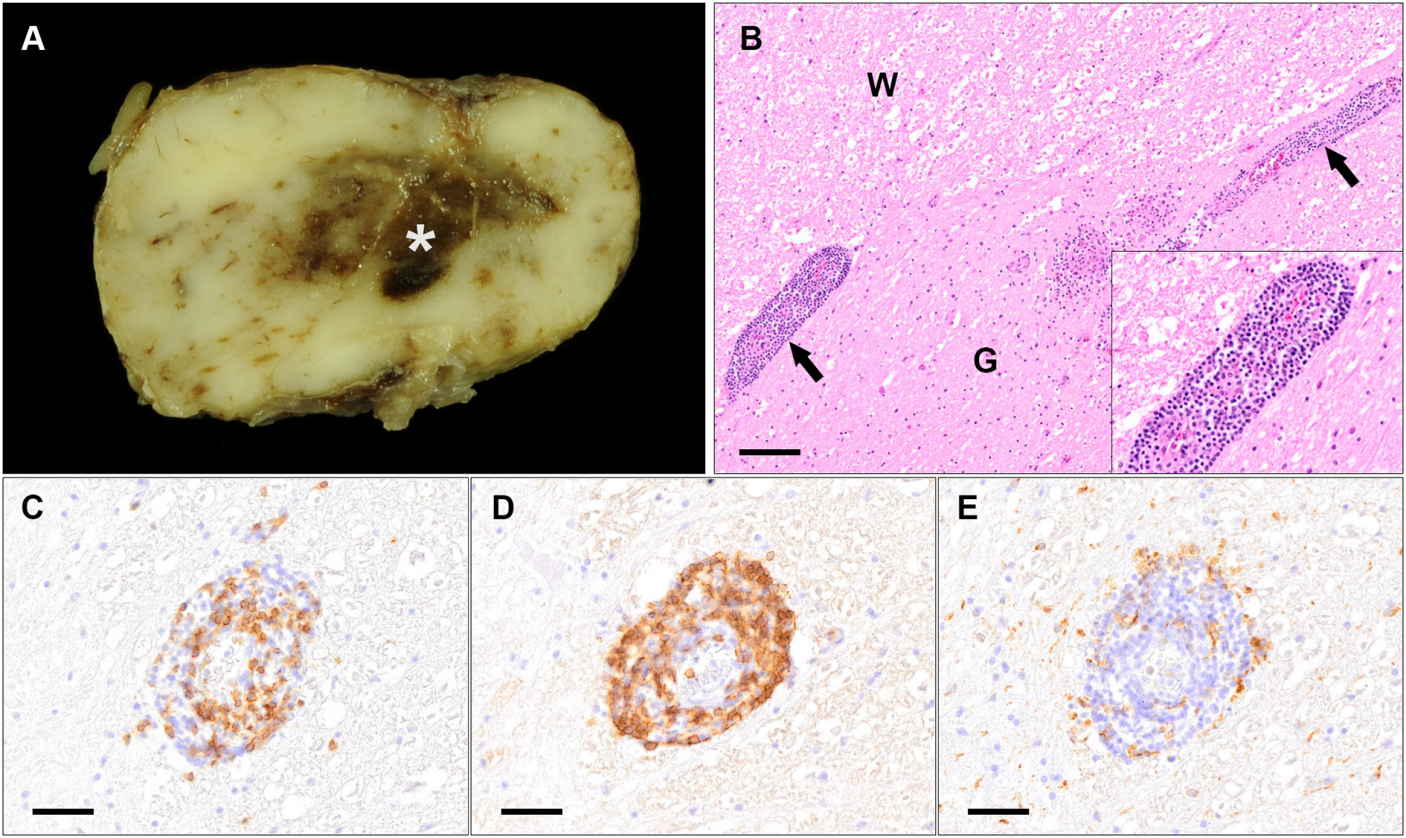
Macroscopic and histopathological findings of a 10.75 year old Australian Shepherd (case 3). **(A)** Macroscopic image of the brain stem displaying focal, severe hemorrhage within the neuroparenchyma (asterisk). **(B)** Histopathology of the spinal cord shows severe, multifocal vasculitis characterized by infiltrating inflammatory cells in the damaged vascular wall and the perivascular space (arrows; insert displays higher magnification of the perivascular infiltrates) at the border between white matter (W) and gray matter (G) (HE stain; scale bar: 100 μm). **(C–E)** Immunohistochemistry for CD3 **(C)**, CD20 **(D)** and CD204 **(E)** shows that the majority of inflammatory cells infiltrating and surrounding damaged vessels are comprised of many **(B–D)** and less T-lymphocytes **(C)**. Only very few infiltrating cells represent CD204-positive macrophages **(E)** (Scale bar: 50 μm).

Necropsy revealed a generalized swelling of the brain with mild herniation of the cerebellar vermis into the *foramen occipitale magnum* as well as herniation of the occipital lobe underneath the *tentorium cerebelli osseum* as indicated in the MRI. Furthermore, there was severe, acute hemorrhage and softening of the neuroparenchyma within the brain stem (Figure 5A).

Histologically, the cerebral cortex, cerebellum, brain stem as well as the spinal cord showed severe, lympho-histiocytic and plasmacytic panencephalomyelitis and meningitis with perivascular edema (Figure 5B). Additionally, severe vasculitis with inflammatory infiltrates in the damaged vascular wall (leukocytoclastic vasculitis) and in the perivascular space was found within the brain and the spinal cord, accompanied by moderate to severe hemorrhage. Spinal ganglia displayed mild to moderate infiltration of lymphocytes and macrophages, too. Additionally, the right eye displayed a mild to moderate, lympho-histiocytic to granulomatous neuritis and perineuritis of the optic nerve.

IHC of the CNS (Figures 5C–E) revealed that the majority of inflammatory cells infiltrating the vascular wall as well as the perivascular space were comprised of B- and T-lymphocytes. B-lymphocytes outnumbered T-lymphocytes in most of the investigated areas. Few infiltrating cells represented CD204-positive macrophages in these regions. However, the number of infiltrating macrophages was variably increased in other areas, where less lymphocytes were visible.

Using IHC and histochemistry, no infectious agent or signs of demyelination were detected.

Additionally, the tracheobronchial lymph node showed severe anthracosis. The bone marrow revealed a dominating myeloid cell population. In lungs, heart, gastrointestinal tract, and CNS no parasites were found.

## Summary of the cases

This case series reports about three dogs with signs of severe forebrain disease, which rapidly progressed, additionally involved the brainstem, and subsequently led to death. Extracranial clinical signs were mild and involved respiratory signs in one dog. MRI examination in two dogs showed generalized swelling of cerebral gray matter and subsequent features of increased intracranial pressure as well as signs of cerebellar and brainstem hemorrhage or herniation. Pathological contrast uptake could not be evaluated, because it was not administered in *post mortem* MRI in case 1, because no MRI was performed due to peracute death in case 2, and because of suspected insufficient intracranial circulation of contrast medium in case 3. Cerebrospinal fluid examination was only performed in case 1 and revealed hemorrhage, and lymphocytic dominance in cell differentiation analysis.

Macroscopically, the brains of two dogs displayed edema of varying degree and cerebellar herniation, and the brains of all dogs displayed hemorrhages occasionally. Microscopically, the main findings comprised lympho-histiocytic inflammation in the brain and/or spinal cord with associated leukocytoclastic and non-leukocytoclastic vasculitis.

An infectious causative agent could not be determined in any of the cases.

## Discussion

Meningoencephalitis of unknown origin (MUO) in dogs is defined as a primary inflammatory brain disease with a so far unknown trigger (2, 3). Clinical signs of focal to multifocal encephalopathy, advanced diagnostic imaging (preferably MRI) revealing focal or multifocal, intraaxial lesions (frequently with increased contrast enhancement), CSF pleocytosis as well as the exclusion of potential causative infectious agents (3), suggest MUO in a clinical setting. However, definitive diagnosis requires histopathological confirmation (3, 15, 26).

MUO subtypes, like GME, NME, NLE, and Greyhound encephalitis, present with distinct histopathological features (12, 13, 16–19). In none of them, CNS vasculitis is a predominant finding (12, 13, 16–19). Vasculitis confined to the CNS is rarely reported (20, 22). Vasculitis is typically generalized or most pronounced in other organs than CNS such as the skin (20, 21) of which approximately 50% of the cases have an idiopathic pathogenesis (27). A focal dermal vasculitis with alopecia is described after subcutaneous rabies vaccination at the side of injection (28, 29). In dogs, the most common form of CNS vasculitis is found in Steroid-responsive meningitis-arteritis (SRMA) (20, 30–32). Acute hemorrhage and/or focal ischemic events secondary to vascular stenosis due to chronic changes of the vascular wall can cause signs of encephalopathy or myelopathy occasionally (20, 33). Histopathologically, SRMA is characterized by fibrinoid necrotizing polyarteritis of small to medium sized predominantly leptomeningeal arteries as well as perivascular and transmural infiltration with lymphocytes, plasma cells, macrophages, and neutrophils (34). Involvement of the arteries in other organs including heart, thyroid, and mediastinum are observed (29). Though, a substantial encephalitis is uncommon and rarely reported (30, 35).

Anecdotal reports about other sterile CNS vasculitis include localized or generalized, fibrinoid necrotizing vasculitis with and without associated ischemic lesions (20, 36), fibrinoid necrotizing changes of leptomeningeal blood vessels with purulent inflammation (20), segmental mononuclear vasculitis of the ventral spinal artery branches in a Miniature Schnauzer (20), and chronic demyelinating vasculitis in a middle-aged Weimaraner (31). The pathological findings of the cases presented in this report are characterized by meningoencephalitis and CNS vasculitis and do not resemble any of these described cases. Here, vasculitis was restricted to the CNS. Mostly small to medium sized blood vessels of the parenchyma as well as the leptomeninx were affected. The changes were asymmetrical, affected both the gray and white matter or only the gray matter and severity ranged from mild to severe depending on the area. The clinical signs of the dogs in this case series were acute, rapidly progressive, and severe. Most likely discontinuity of the inflamed vascular walls led to break down of the blood-brain barrier and generalized edema and hemorrhage (37) as observed macroscopically and histologically. This led to a quick increase of intracranial pressure, partly with caudal cerebellar herniation; as a consequence, centrally controlled vital functions most likely got compromised (38). If the acute and fatal outcome of these cases is representative for the here presented lympho-histiocytic meningoencephalitis with CNS vasculitis is unclear, as necropsy was one of the inclusion criteria in this case series, which might cause a clinical bias. Therefore, no statement can be made about the clinical prognosis or about specific therapy recommendations.

Clinical signs in MUO are mostly restricted to neurological abnormalities; signs of systemic disease are rare (1). In this case series only case 1 presented with coughing while no other extracranial sings were evident *ante mortem*. Respiratory signs could have been indicative for a triggering respiratory infection or might have been unrelated to the encephalitis. However, necropsy and histopathology did not reveal signs of respiratory or systemic disease. In case 3, hyperthermia, tachycardia, and hemorrhagic diarrhea were suspected to be secondary to prolonged seizure activity not primarily due to the disease itself.

Interpretation of CSF examinations were restricted. Severe contamination with blood prevented adequate leukocyte cell count. It might be caused by iatrogenic vessel damage during CSF tap or might reflect intracranial hemorrhage (39). The latter seems more likely, as MRI was indicative of multifocal caudal fossa hemorrhage (40).

For the here presented meningoencephalitis with CNS vasculitis no infectious agents could be detected. However, this does not necessarily exclude an infectious etiology. This could be due to the fact that a potentially causative infectious agent was no longer detectable at the time of investigation [“hit and run theory” (41)]. Furthermore, the risk of false-negative results in the performed investigations needs to be taken into account as well. Pathogenetically, similar lesions can be triggered directly by an infectious agent or could be the result of immune-mediated pathogen-triggered mechanisms such as molecular mimicry or epitope-spreading [reviewed by Pederson (42)]. Moreover, so far unknown pathogens not discovered by immunohistochemistry need to be considered, since several recent investigations using next generation sequencing have revealed new, so far unknown etiologies (43, 44).

To the best of the author's knowledge, this is the first case series describing clinical signs, diagnostic imaging and histopathological findings of an acute, progressive, fatal non-suppurative meningoencephalitis with vasculitis restricted to the CNS. It is proposed to consider the described changes of the three dogs as a new subtype of MUO with CNS vasculitis as the histopathological distinguishing feature.

## Data availability statement

The raw data supporting the conclusions of this article will be made available by the authors, without undue reservation.

## Ethics statement

Ethical review and approval was not required for the animal study because retrospective case report. Written informed consent was obtained from the owners for the participation of their animals in this study.

## Author contributions

IZ performed and interpreted results of necropsy and histopathology and drafted manuscript. JR performed and interpreted findings of diagnostic imaging and finalized manuscript. FS performed and supervised anesthesia, took care for patients, and finalized manuscript. WB interpreted results of necropsy and histopathology and finalized manuscript. AT interpreted findings of clinical and diagnostic imaging examination and finalized manuscript. JN performed and interpreted findings of clinical and diagnostic imaging examinations, drafted, and finalized manuscript. All authors agree on authorship and publication of the manuscript. All authors contributed to the article and approved the submitted version.

## Funding

This Open Access publication was funded by the Deutsche Forschungsgemeinschaft (DFG, German Research Foundation) - 491094227 Open Access Publication Costs and the University of Veterinary Medicine Hannover, Foundation.

## Conflict of interest

The authors declare that the research was conducted in the absence of any commercial or financial relationships that could be construed as a potential conflict of interest.

## Publisher's note

All claims expressed in this article are solely those of the authors and do not necessarily represent those of their affiliated organizations, or those of the publisher, the editors and the reviewers. Any product that may be evaluated in this article, or claim that may be made by its manufacturer, is not guaranteed or endorsed by the publisher.
